# Tensile Performance Mechanism for Bamboo Fiber-Reinforced, Palm Oil-Based Resin Bio-Composites Using Finite Element Simulation and Machine Learning

**DOI:** 10.3390/polym15122633

**Published:** 2023-06-09

**Authors:** Wenjing Wang, Yuchao Wu, Wendi Liu, Tengfei Fu, Renhui Qiu, Shuyi Wu

**Affiliations:** College of Transportation and Civil Engineering, Fujian Agriculture and Forestry University, Fuzhou 350108, China

**Keywords:** bamboo fibers, bio-resin, bio-composites, finite element simulation, machine learning

## Abstract

Plant fiber-reinforced composites have the advantages of environmental friendliness, sustainability, and high specific strength and modulus. They are widely used as low-carbon emission materials in automobiles, construction, and buildings. The prediction of their mechanical performance is critical for material optimal design and application. However, the variation in the physical structure of plant fibers, the randomness of meso-structures, and the multiple material parameters of composites limit the optimal design of the composite mechanical properties. Based on tensile experiments on bamboo fiber-reinforced, palm oil-based resin composites, finite element simulations were carried out and the effect of material parameters on the tensile performances of the composites was investigated. In addition, machine learning methods were used to predict the tensile properties of the composites. The numerical results showed that the resin type, contact interface, fiber volume fraction, and multi-factor coupling significantly influenced the tensile performance of the composites. The results of the machine learning analysis showed that the gradient boosting decision tree method had the best prediction performance for the tensile strength of the composites (*R*^2^ was 0.786) based on numerical simulation data from a small sample size. Furthermore, the machine learning analysis demonstrated that the resin performance and fiber volume fraction were critical parameters for the tensile strength of composites. This study provides an insightful understanding and effective route for investigating the tensile performance of complex bio-composites.

## 1. Introduction

Sustainable bio-composites have attracted extensive attention for reducing carbon emissions by replacing traditional synthetic fiber-reinforced, petroleum-based resin composites. Plant fibers, such as hemp fiber, palm leaf fiber, wood fiber, and bamboo fiber (BF) [1], have the advantages of abundant resources, low price, renewability, degradability, and high specific strength and modulus [2,3]. Among them, BFs have outstanding mechanical properties and the potential to be applied in green composites. The thermosetting resin made from vegetable oils has become an interesting topic in sustainable materials. Palm oil (PO) has the lowest price and the largest production among plant oils in the world, and some developed PO-based resins (PORs) have shown high strength, good fatigue resistance, biodegradability, and low environmental impact [4,5,6]. Thus, by combining the advantages of BFs and PORs, the BF/POR bio-composite was prepared and expected to be widely used in construction, automobile parts, furniture, and packaging. A profound understanding of the mechanical properties is necessary for the design and application of this composite. However, the tensile performance of the composite varies largely due to the high variance in structural and mechanical parameters of plant fibers and bio-based resins, random meso-structures of discontinuous fibers in composites, and complex interface properties between fibers and resins. Thus, it is difficult to determine the mechanism for the tensile properties of this composite, limiting its potential for wide application.

Experimental methods such as mechanical testing, scanning electron microscopy, atom force microscopy, and nano-indentation have been used to characterize the mechanical properties, microstructure, interfacial morphology, and microscopic stiffness distribution of composites [1,4]. However, it is difficult to investigate various failure modes such as matrix failure, fiber fracture, and interfacial debonding using experimental methods [7,8]. Moreover, it is also difficult to systematically investigate the effect of multiple factors on the mechanical properties of plant fiber-reinforced composites [9,10,11,12,13]. 

Numerical simulation can show stress distributions in the microstructures of composite materials, revealing the mechanical mechanism and accelerating material optimization. Finite element simulation has been used to obtain stress distributions and analyze failure modes in the representative volume element (RVE) of composites [14,15,16]. However, finite element simulation has mainly been used for composites with uniform, continuous, and periodic fiber structures [17,18]. Therefore, an effective method should be further developed to model the BF/POR bio-composites with distinctive microstructures, i.e., the long discontinuous and uniformly orientated bamboo fibers distributed in the composites [19].

Machine learning (ML) methods are powerful tools for predicting the mechanical properties of composites with various parameter combinations, identifying the most important factors [20,21,22], and investigating the nonlinear relationship between multiple factors and the mechanical properties of composites [23]. The support vector machine, decision tree, random forest, gradient boosting, artificial neural network, extreme gradient boosting, and other ML methods have been applied to predict and analyze the strength of concrete in the marine environment [24], the compressive strength of high-performance concrete [25], the interfacial strength of steel and carbon fiber-reinforced composites [26], the strength of concrete with microencapsulated phase change material [27], and the strength of concrete-filled steel tubes [28]. Numerical simulation obtains material performance by building models for the RVE and calculating the stress and strain based on physical principles. Meanwhile, the ML predicts material properties by learning the relation between parameters and properties of materials based on data. Thus, the prediction using ML methods is much quicker, which is necessary to efficiently investigate the performance of materials with multiple parameters and large parameter ranges.

The size of the data set for ML is usually small due to the time and economic costs required to collect samples for a specific material, significantly limiting the performance of ML models. Thus, integrated ML methods have attracted attention for improving the forecast accuracy when using small volume samples by aggregating multiple base learners using two main strategies, i.e., the bagging and boosting strategies. The bagging strategy can reduce prediction variance by repeating the training and calculating the average of multiple forecast results [29]. The boosting strategy can decrease prediction errors by iteratively compensating the prediction errors and calculating the sum of multiple analysis results in series [30]. Based on the decision tree (DT) method, the integrated random forests (RF) method was established according to the bagging strategy. The gradient boosting decision tree (GBDT), extreme gradient boosting (XGBoost), and categorical feature-supported gradient boosting (CatBoost) methods were established according to the boosting strategy [31,32,33].

This study investigated the tensile properties of BF/POR bio-composites using parametric modeling, finite element simulation, and ML methods including DT, RF, GBDT, XGBoost, and CatBoost. The effects of resin type, interface property, fiber volume fraction, and fiber length and diameter on the tensile properties of composites were investigated. The tensile properties of composites with different material parameters were predicted, and the most important parameter for tensile strength was identified. This study provides an effective approach for investigating the tensile properties of plant fiber-reinforced bio-composites and guidance for optimizing these composites.

## 2. Materials and Methods

### 2.1. Preparation of Bamboo Fiber-Reinforced, Palm Oil-Based Resin Composites

The preparation and measurement of three kinds of PO-based resins (PORs) and bamboo fiber (BF)-reinforced composites were previously reported in our study [34]. Briefly, three PORs, namely POFA_EA_EM, POFA_EA_GM, and POFA_EA_TM, were synthesized from palm oil (PO) using transesterification followed by a reaction with three natural polyphenols. First, PO fatty acid–ethyl acrylamide (POFA-EA) was synthesized from PO and *N*-(2-hydroxyethyl) acrylamide. Then, the POFA-EA was further reacted with three natural phenolic cross-linkers, i.e., eugenol–methacrylate (EM), methyl gallate–methacrylate (GM), and tannic acid–methacrylate (TM). The resultant resins were represented by POFA_EA_EM, POFA_EA_GM, and POFA_EA_TM, respectively. The BF-reinforced composites were prepared by carding original BFs into unidirectional fiber mats, coating the mats with resin, and hot-pressing the mats. The average length and diameter of the BFs were 22.81 mm and 150 μm, respectively. The composites exhibited a key feature of regularly arranged discontinuous long fibers. Tensile tests on the composites were carried out using an Instron 3365 universal testing machine (Instron, Norwood, MA, USA) at a crosshead speed of 10 mm/min. Each measurement was repeated 6 times.

### 2.2. Finite Element Simulation

The improved sequential random perturbation method was used to establish a two-dimensional model of the randomly distributed fibers in the cross-section of the composites. The initial two-dimensional model was established according to the parameters including area size, fiber radius, fiber volume fraction, and the minimum distance between fibers. Each fiber center was randomly moved to generate the final two-dimensional model of the composites. Python software was used to generate three-dimensional discontinuous fiber clusters. The distance between fiber ends was set as 150 μm. Boolean operations were carried out to build the fiber part and resin part in an RVE. The size of the RVE was set as 1 mm × 1 mm × 5 mm The C3D6 element was used to mesh the fiber and resin with a mesh size of approximately 4 μm. The BFs and POR were simulated as linear elastic material and plastic material, respectively.

### 2.3. Machine Learning (ML) Method

The dataset containing composite tensile strength for the ML analysis included 82 samples obtained using finite element simulation. The output value was the tensile strength (*TS*) of the composites, while the input features consisted of BF volume fraction (*VF*), fiber length (*L*), fiber diameter (*D*), the ratio of length to diameter (*L/D*), the fiber reinforcement coefficient (*RI = VF* × *L/D*), resin failure stress (*R*_stress_), and resin failure strain (*R*_strain_). The interface between the fiber and resin was set as a combined condition for this dataset. 

The dataset was randomly divided into training data and test data with a ratio of 7:3. Then five ML models including DT, RF, GBDT, XGBoost, and CatBoost were developed to predict the tensile strength of the composites and analyze the importance of each feature. For each ML model, ten cross-validations were performed, and the average of the cross-validation results was used to assess the performance of the ML models with different datasets. The performance of the ML models was evaluated using three statistical indicators including the mean absolute error (MAE), root mean square error (RMSE), and coefficient of determination (*R*^2^). These metrics were obtained as follows (Equations (1)–(3)):

(1) $$\mathrm{MAE}=\frac{1}{n}\overset{n}{\sum }|y-\hat{y}|$$

(2) $$\mathrm{RMSE}=\sqrt{\frac{1}{n}\overset{n}{\underset{i=1}{\sum }}{\left(y-\hat{y}\right)}^{2}}$$

(3) $$R^{2}=1-\frac{\sum^{n}{\left(y-\hat{y}\right)}^{2}}{\sum^{n}{\left(y-\bar{y}\right)}^{2}}$$

where n is the number of observations in the dataset, y is the observed output, $\bar{y}$ is the average observed output, and $\hat{y}$ is the predicted output of the ML models.

### 2.4. DT Algorithm

The DT algorithm predicts the output by recursively dividing the data into smaller groups based on conditions and finally classifying data samples. To enhance the ML model, the DT algorithm can be used as a base learner in integrated ML models.

### 2.5. RF Algorithm

The RF algorithm randomly selects training samples and input features to establish various training datasets for different decision trees. The final output value of a RF is obtained by averaging the results of all decision trees as follows (Equation (4)):

(4) $$\hat{Y}=\frac{1}{N}\overset{N}{\underset{i=1}{\sum }}\;{\hat{Y}}_{i}$$

where $\hat{Y}$ is the final predicted value of the RF model, *N* is the number of basic DT models in a given RF model, and ${\hat{Y}}_{i}$ is the predicted value of the ith DT model. This “bagging” strategy can reduce the variance and avoid overfitting. 

### 2.6. GBDT Algorithm

The GBDT algorithm progressively generates new base learners and trains them based on the residual error of the previous model to continuously reduce the prediction error. The final result of this model is obtained by combining base learners with different weights as follows (Equation (5)):

(5) $$f_{M}(x)=\sum_{m=1}^{M}T\left(x;\theta_{m}\right)$$

where M denotes the number of basic models, $T\left(x;\theta_{m}\right)$ is the basic tree model, x represents the splitting condition of nodes, $\theta_{m}$ represents all the parameters in the basic model such as random state and the parameters for branch cutting, and $f_{M}(x)$ is the final output. This “boosting” strategy can reduce the prediction deviation.

### 2.7. XGBoost Algorithm

The XGBoost algorithm uses the same “boosting” strategy as the GBDT algorithm mentioned above. XGBoost uses second-order Taylor expansion for the loss function to promote the model convergence by obtaining a quadratic convex function, while GBDT only considers the first-order derivative. Furthermore, XGBoost includes an improved method to determine the splitting node based on the objective functions. The objective function in the XGBoost model before and after splitting a node is shown in Equations (6) and (7), respectively. The best splitting node is identified when the largest difference between the two objective functions is obtained (Equation (8)).

(6) $$Obj_{1}=-\frac{1}{2}\left[\frac{{\left(G_{L}+G_{R}\right)}^{2}}{H_{L}+H_{R}+\lambda }\right]+\gamma$$

(7) $$Obj_{2}=-\frac{1}{2}\left[\frac{G_{L}^{2}}{H_{L}+\lambda }+\frac{G_{R}^{2}}{H_{R}+\lambda }\right]+2\gamma$$

(8) $$Gain=Obj_{1}-Obj_{2}=\frac{1}{2}\left[\frac{G_{L}^{2}}{H_{L}+\lambda }+\frac{G_{R}^{2}}{H_{R}+\lambda }-\frac{{\left(G_{L}+G_{R}\right)}^{2}}{H_{L}+H_{R}+\lambda }\right]-\gamma$$

where $Obj_{1}$ and $Obj_{2}$ are the objective functions in the XGBoost model before and after node splitting, respectively. Gain is the change in the objective function after splitting, G represents the sum of the first derivatives of all leaf nodes, H represents the sum of the second derivatives of all leaf nodes, and the subscripts L and R represent the left and right branches, respectively. $\frac{{\left(G_{L}+G_{R}\right)}^{2}}{H_{L}+H_{R}+\lambda }$ is the score before branching, $\frac{G_{L}^{2}}{H_{L}+\lambda }$+$\frac{G_{R}^{2}}{H_{R}+\lambda }$ is the score sum of two sub-trees after branching, and λ and γ are used to control the complexity of the model. 

### 2.8. CatBoost Algorithm

The CatBoost algorithm is another integrated ML model that uses the “boosting” strategy [35]. In other boosting algorithms, the true value of a category feature is calculated as the average of the true values of this category feature for all samples (Equation (9)):

(9) $$x_{i,\;k}=\frac{\sum_{j=1}^{n}\left[x_{j,\;k}=x_{i,\;k}\right]\cdot Y_{j}}{\sum_{j=1}^{n}\left[x_{j,\;k}=x_{i,\;k}\right]}\;\mathrm{where}\;\left[x_{j,\;k}=x_{i,\;k}\right]=\left\{\begin{array}{l} 1,x_{j,\;k}=x_{i,\;k}\\ 0,\;x_{j,\;k}\neq x_{i,\;k} \end{array}\right.$$

where $X_{i}=\left(x_{i,\;1},\ldots ,x_{i,\;m}\right)$ is the vector including m features, $Y_{i}$ is the true label value of the ith sample for a dataset of $D={\left\{\left(X_{i},Y_{i}\right)\right\}}_{i=1\cdots n}$, and $x_{i,\;k}$ is the *k*th feature of the ith training sample. This approach may lead to conditional shifts [36].

To solve the problem of calculating the true value for a category feature using boosting algorithms, CatBoost sorts the samples randomly and calculates the *k*th category feature of the *p*th data in the sorted dataset, i.e., $x_{\sigma_{p,\;k}}$, according to Equation (10).

(10) $$x_{\sigma_{p,\;k}}=\frac{\sum_{j=1}^{p-1}\left[x_{\sigma_{j,\;k}}=x_{\sigma_{p,\;k}}\right]Y_{\sigma_{j}}+\beta P}{\sum_{j=1}^{p-1}\left[x_{\sigma_{j,\;k}}=x_{\sigma_{p,\;k}}\right]+\beta }$$

where P is the prior value and β is the weight of the prior value. CatBoost uses a greedy strategy to consider new features produced by combining current features, thus improving prediction performance.

## 3. Results and Discussion

### 3.1. Preparation and Numerical Model of the Composites

**Figure 1:**
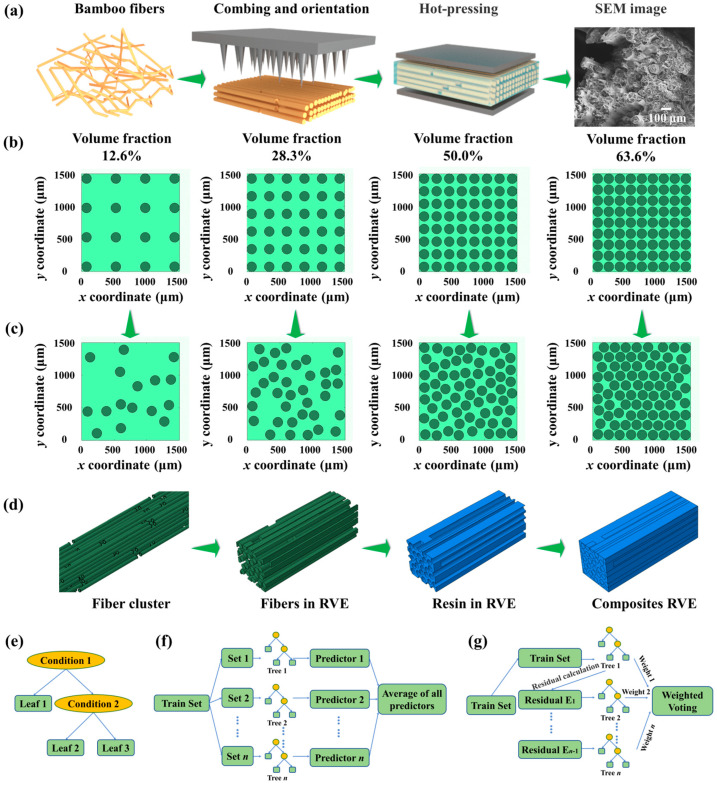
Preparation, numerical model, and ML models of the composites. (**a**) The preparation process and SEM image of the composites. The fiber distribution in the cross-section of the composites with different fiber volume fractions at (**b**) the initial stage and (**c**) the final stage. (**d**) The 3D models for fiber clusters, fibers in the RVE, the resin in the RVE, and the RVE of composites. Structural sketch of the ML models including (**e**) DT, (**f**) RF, and (**g**) GBDT.

Figure 1a shows the preparation processes for the composites, including combing BFs into oriented fiber clusters, pouring POR onto the bamboo fiber clusters, and hot-pressing [34]. The SEM image shows the features of the composite, that is, the discontinuous long fibers were regularly arranged. To establish the RVE of the numerical model for the composites, the two-dimensional structure of cross-sections with oriented fibers distribution was built according to the various volume fractions of fibers. The circles stand for the cross-sections of fibers (Figure 1b). Then, the circles randomly migrated to obtain the two-dimensional sections with randomly distributed fibers. A minimum distance between the circle centers was set to avoid overlap between fibers (Figure 1c). Based on the two-dimensional structures of the cross-sections, three-dimensional fiber clusters were established by stretching the circles along the direction perpendicular to the two-dimensional cross-sections. The fiber lengths and interruption distances between the ends of the fibers were set to reflect the discontinuity of fibers. Then, the RVEs for the composites were established based on the three-dimensional fiber clusters using Boolean operations (Figure 1d). The simulation results provided the dataset for ML analysis (Figure 1e–g). 

### 3.2. Experiment and Simulation of PORs

**Figure 2:**
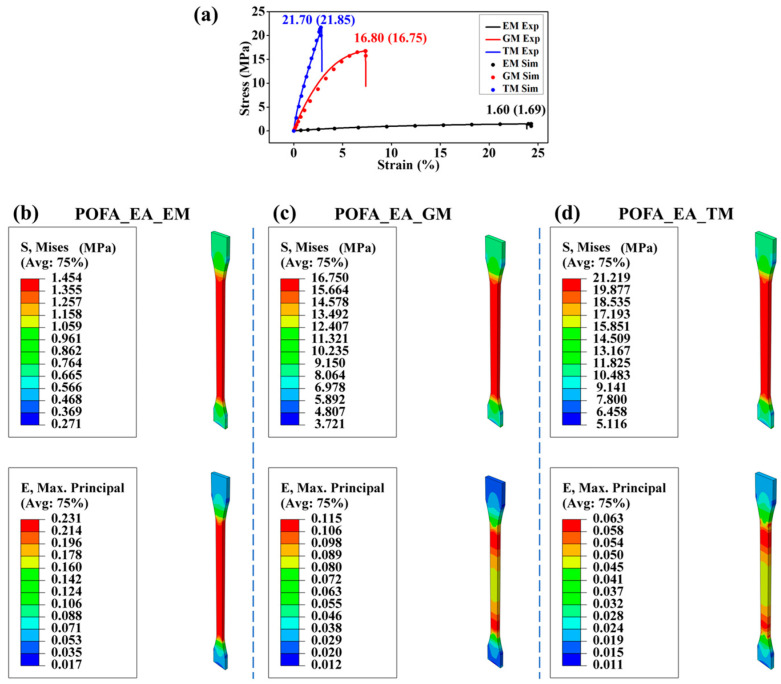
Tensile properties of three kinds of resins. (**a**) Comparison of the tensile curves of resins from experiments and simulations, where EM Exp, GM Exp, and TM Exp refer to the experimental results for the POFA_EA_EM, POFA_EA_GM, and POFA_EA_TM resins, respectively; and EM Sim, GM Sim, and TM Sim refer to the simulation results for the POFA_EA_EM, POFA_EA_GM, and POFA_EA_TM resins, respectively. The stress and strain distributions in (**b**) POFA_EA_EM, (**c**) POFA_EA_GM, and (**d**) POFA_EA_TM resins.

Figure 2 shows the tensile stresses obtained from simulations and experimental results for three kinds of PORs, which were developed and measured in our previous study [34]. The results indicate the accuracy of the simulation and the significant difference in the tensile properties among the resins. The tensile strength of the POFA_EA_TM composite was much higher than the others, which may be owed to the rigid aromatic structure of TM and the high cross-linking density of the resin. The POFA_EA_EM composite had a much higher elongation at the break due to fewer C=C bonds and benzene rings in EM [37]. The mechanical parameters of the resin in the subsequent composite models were set based on the above simulations of the PORs.

### 3.3. Effect of Resin Type on the Tensile Properties of Composites

**Figure 3:**
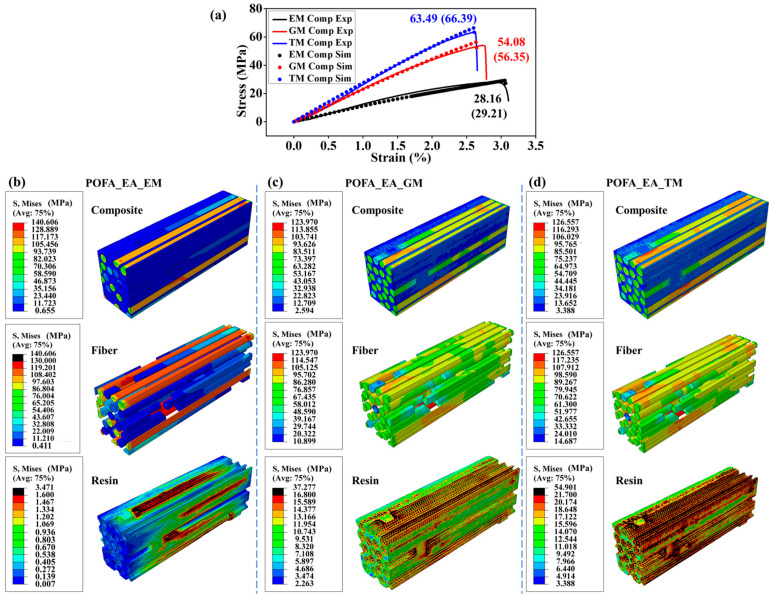
Effect of resin type on composite properties. (**a**) Tensile curves for the three composites from simulations and experiments, where EM Comp Exp, GM Comp Exp, and TM Comp Exp refer to the experimental results for the POFA_EA_EM composite, POFA_EA_GM composite, and POFA_EA_TM composite, respectively; and EM Comp Sim, GM Comp Sim, and TM Comp Sim refer to the simulation results for the POFA_EA_EM composite, POFA_EA_GM composite, and POFA_EA_TM composite, respectively. Stress distributions in the components and the composites with (**b**) POFA_EA_EM, (**c**) POFA_EA_GM, and (**d**) POFA_EA_TM resins. The fiber volume fraction was set at 50%, and the interface adhesion was set to an ideally combined mode.

Figure 3a shows that the numerical results of the stress–strain curves for the composites with three resins agree well with the experimental ones, thus suggesting the accuracy of the simulations. The tensile properties of three composites were demonstrated using the numerical stress and strain distributions of composites and their components (Figure 3b–d). Because of the small modulus and large elongation at the break of the POFA_EA_EM resin, the main failure mode of this composite was the tensile fracture of BFs. Furthermore, due to the significant modulus difference between the POFA_EA_EM resin and BFs, the failure at the interface between BF and resin occurred by shear force (Figure 3b). For the composites with the POFA_EA_GM and POFA_EA_TM resins, the failure occurred at the resin matrix caused by the combination of tensile and shear forces (Figure 3c,d).

The simulation results indicate the significant influence of fiber continuity on the composite performance. The continuous fibers in the composite RVE mainly bear the tensile force, and the discontinuous fibers majorly withstand the shear force transferred from the resin matrix. For the composite with the POFA_EA_EM resin, because of the low shear stress transferring from the low-modulus resin matrix, the stress on the discontinuous fibers was only about 15% of the continuous ones (Figure 3b). The POFA_EA_GM and POFA_EA_TM resins have relatively higher moduli; therefore, the shear force transferred from the resin matrix to the discontinuous fiber was larger, and there was no significant difference in the stress between the discontinuous and continuous fibers (Figure 3c,d).

The simulations proved the micro-mechanism for the tensile strength of the composites. Resin with higher strength, such as POFA_EA_TM, can bear higher stress. Furthermore, higher shear stress can be transferred to the discontinuous fibers from resin with a higher modulus to improve the bearing efficiency of fibers. Moreover, the shear force at the interface was mild when the moduli of the resin and fiber were comparable, which avoided premature shear failure of the resin at the interface and improved the bearing efficiency of the resin. 

### 3.4. Effect of Interface Properties on Tensile Properties of Composites

**Figure 4:**
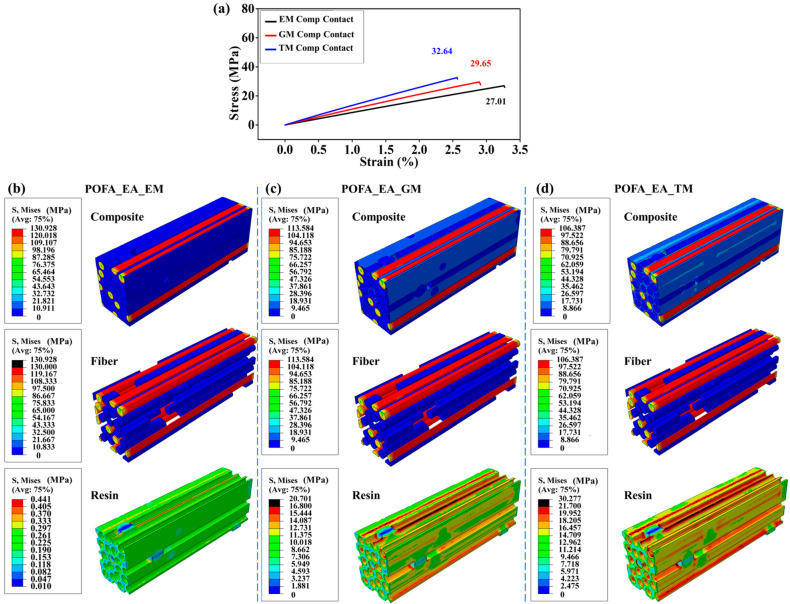
Numerical simulation results for composite performance with a contact interface. (**a**) Tensile curves for three composites. The stress distributions in the components and the composites with (**b**) POFA_EA_EM, (**c**) POFA_EA_GM, and (**d**) POFA_EA_TM resins. The fiber volume fraction was set at 50%, and the interface property was set to the contact mode.

To investigate the influence of interface properties on composite performance, the fiber–resin interface was set from the combined mode (Figure 3a) to the contact mode (Figure 4a). The tensile strength of the composites with POFA_EA_EM, POFA_EA_GM, and POFA_EA_TM resins decreased by 4.1%, 45.2%, and 48.6%, respectively, as the interface property changed from the combined mode to the contact mode. This result was attributed to the lower shear force transferred from resin to the discontinuous fibers by the weak interface bonding (Figure 4b–d). Moreover, the influence of interface bonding was more significant for the composites with high-modulus resins. The tensile strength of the composite with a high-modulus resin, i.e., the POFA_EA_TM resin, significantly decreased by about 50% and the stress of the composites with discontinuous fibers decreased by 89% as the interface property changed from the combined mode to the contact mode. Meanwhile, for the POFA_EA_EM resin, i.e., a relatively low-modulus resin, the decrease in strength and stress distribution in the composite was not significant. These results indicate that the fiber–resin interface adhesion is critical for composite performance with high-modulus resins. The interface property also affected the failure mode of the composite. As the interface property was set to the contact mode, the tensile fracture of fibers was the main failure mode for the composite with the POFA_EA_EM resin, while the resin tensile failure was the dominant failure mode for the composite with the POFA_EA_GM and POFA_EA_TM resins. 

### 3.5. Effect of Fiber Volume Fraction on Tensile Properties of Composites

**Figure 5:**
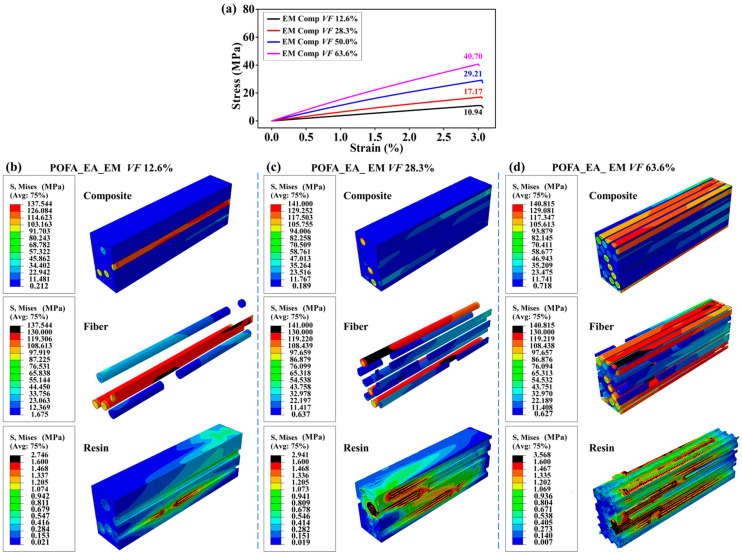
Tensile performance of BF-reinforced POFA_EA_EM resin composites with different fiber volume fractions. (**a**) Tensile curves for the composites. The stress distributions in the components and composites with a fiber volume fraction of (**b**) 12.6%, (**c**) 28.3%, and (**d**) 63.6%. The interface property was set to a combined mode.

**Figure 6:**
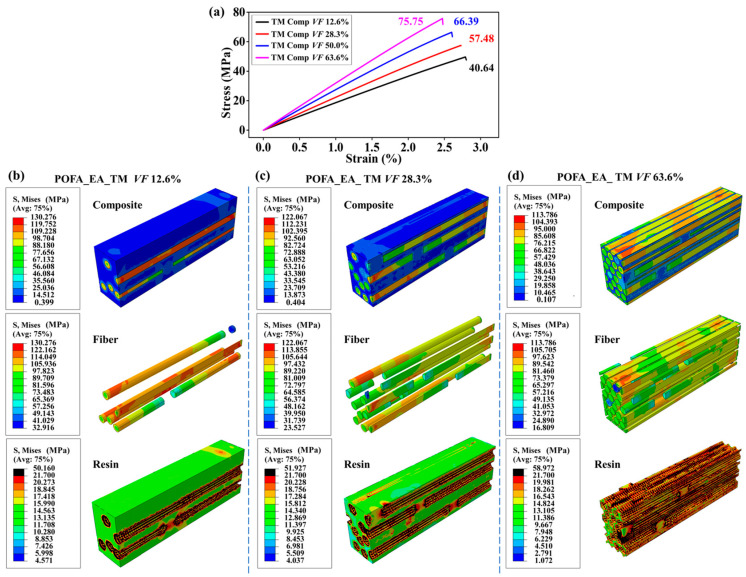
Tensile performance of BF-reinforced POFA_EA_TM resin composites with different fiber volume fractions. (**a**) Tensile curves for the composites. The stress distributions in the components and composites with a fiber volume fraction of (**b**) 2.6%, (**c**) 28.3%, and (**d**) 63.6%. The interface property was set to a combined mode.

As the fiber volume fraction increased from 12.6 to 63.6%, the tensile strength of the composites with the POFA_EA_EM and POFA_EA_TM resins increased by 272.0% and 86.4%, respectively (Figure 5a and Figure 6a). With the increase in the fiber volume fraction, the low-modulus POFA_EA_EM resin was replaced with BFs, significantly improving the tensile strength of the composites. Moreover, the interface area with large stress distributions between the BFs and resin increased with the increase in the fiber volume fraction, improving the bearing efficiency of the resin matrix and the composite tensile performance (Figure 5b−d). However, the fiber fraction and interface area had less impact on the composites with the high-modulus POFA_EA_TM resin matrix (Figure 6b−d).

### 3.6. Predictive Performance of Composites using ML Models

**Figure 7:**
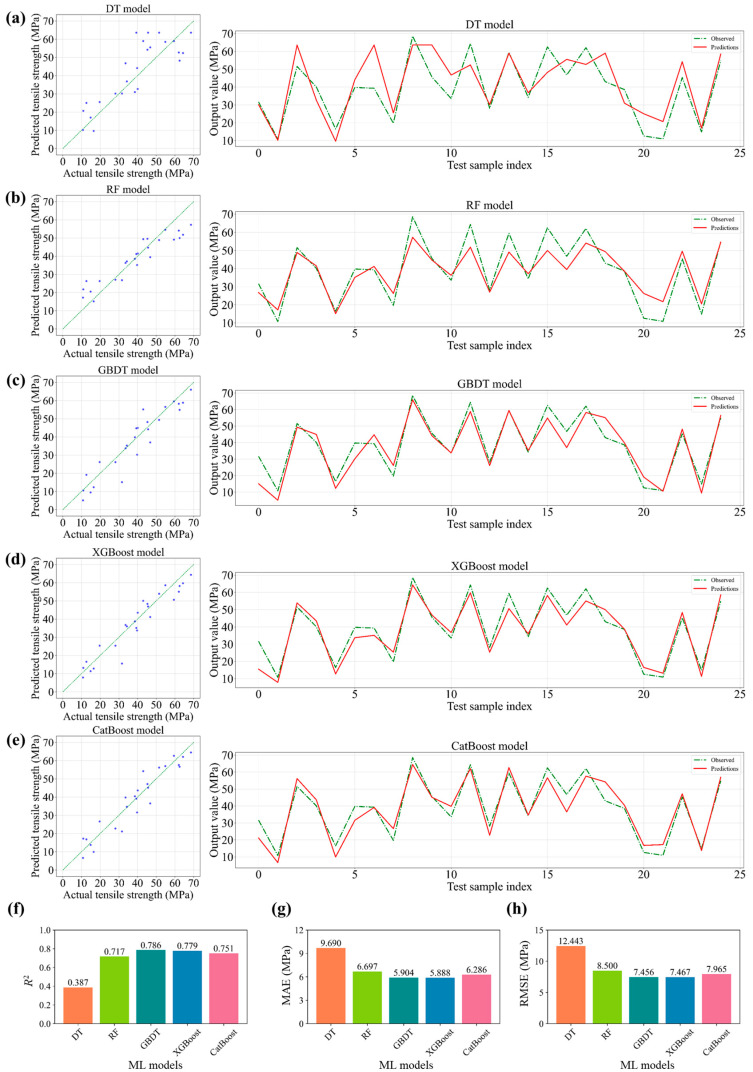
The prediction performance of five ML models. The tensile strength of the composites from numerically observed values and predicted values obtained using (**a**) DT, (**b**) RF, (**c**) GBDT, (**d**) XGBoost, and (**e**) CatBoost. The values for (**f**) *R*^2^, (**g**) MAE, and (**h**) RMSE from the five ML models.

Five ML models, namely DT, RF, GBDT, XGBoost, and CatBoost, were used to forecast the tensile strength of the composites based on a dataset from the simulations. Table 1 displays the characteristics of the dataset, consisting of seven input variables, i.e., *VF*, *L*, *D*, *L/D*, *RI = VF* × *L/D*, *R*_stress_, and *R*_strain_, and one output variable, i.e., *TS*. The deviations between the true and predicted values for the test dataset samples are shown in Figure 7a−e. The results demonstrate that all five models were able to learn the non-linear coupling relationships among the features and predict the tensile strength. Figure 7f−h shows the quantitative analysis of the performance of the ML models. GBDT performed the best with the highest *R*^2^ value (0.786) and the lowest MAE and RMSE values (5.904 and 7.456 MPa, respectively). The results also indicate that the integrated ML models had better performance than the basic DT model for predicting the tensile strength of the composites. The non-excellent prediction accuracy of the ML models was attributed to the small dataset size and the deviations in the numerical simulations, which were caused by the complexity and randomness of the composite materials. 

### 3.7. Feature Importance

**Figure 8:**
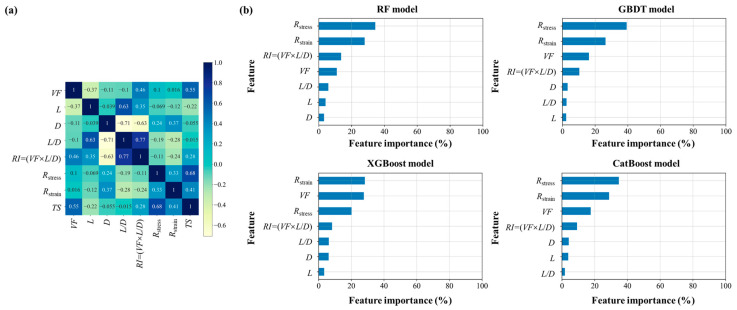
Feature importance analysis. (**a**) Pearson correlation map for the seven input features and one output value. (**b**) Feature importance analysis of four ensemble learning models.

The matrix heat map for the input and output variables shows that the fiber volume fraction and resin failure stress have a high correlation with the composite tensile strength, with correlation coefficients of 0.55 and 0.68, respectively (Figure 8a). Figure 8b shows the relative importance of the features for the prediction of the four integrated learning models. The results indicate that *R*_stress_ and *R*_strain_ were the most important feature for the RF, GBDT, and CatBoost models, which aligns with the effect of resin properties on the composite tensile strength due to the better stress-transferring ability between the discontinuous fibers and the larger stress distributions in resin. Moreover, for the XGBoost model, the *VF* for the fibers was an important structural feature in the current range of parameters. 

## 4. Conclusions

This study found that the finite element simulation was effective for investigating the stress and strain distributions and failure mechanism of the BF/POR composites. The composite tensile performance was significantly influenced by parameters such as resin type, contact interface, fiber volume fraction, and multi-factor coupling. Machine learning methods based on numerical data were appropriate for predicting tensile properties and analyzing feather importance for tensile strength. Using small datasets for finite element simulation, the integrated ML models including RF, GBDT, XGBoost, and CatBoost showed much better prediction ability than the basic DT model. The GBDT model showed the best prediction performance with an *R*^2^ of 0.786. The resin properties and fiber volume fraction were critical parameters for the tensile performance within the current parameter ranges. This study provides an insightful understanding of the simulation, prediction, and optimization of plant fiber-reinforced bio-composites on their tensile properties. In the future, it is worth systematically investigating the mechanical properties of composites under complex loads including tension, shear, and torsion.

## Figures and Tables

**Table 1:** Characteristics of the dataset.

Attribute	*VF* (%)	*L* (μm)	*D* (μm)	*L/D*	*RI*	*R*_stress_ (MPa)	*R*_strain_	*TS* (MPa)
Count	82	82	82	82	82	82	82	82
Mean	40.98	3939.02	207.32	23.09	916.26	15.30	0.10	38.670
Standard Deviation	19.66	1589.69	82.07	14.75	786.05	6.77	0.06	16.305
Minimum	10.00	1000.00	100.00	6.67	66.67	3.35	0.03	9.662
25%	30.00	2000.00	100.00	13.33	400.00	10.61	0.03	26.656
50%	50.00	4000.00	200.00	20.00	666.67	14.95	0.07	39.376
75%	50.00	6000.00	300.00	30.00	1200.00	21.22	0.15	50.306
Maximum	70.00	6000.00	300.00	60.00	4200.00	31.83	0.23	68.799

## Data Availability

The data presented in this study are available on request from the corresponding author.
